# Supplementary material for: Efficacy of Stromal Vascular Fraction Treatment for Knee Osteoarthritis: A Single-Arm Experimental Trial
Source: Biomedicines. 2025 Nov 28;13(12):2913. doi: 10.3390/biomedicines13122913 (PMC12731140; doi:10.3390/biomedicines13122913)
Supplement: Supplementary file 1 [file biomedicines-13-02913-s001.zip › biomedicines-3965640-supplementary.pdf]

Supplementary material

S1. Informed consent

CONSENTIMENT INFORMAT PER A REALITZACIÓ, APLICACIÓ I / O DIPÒSIT DE CELSTEM®

Llegiu detingudament la informació present en aquest document abans de donar el seu consentiment al tractament amb CelStem®.

INTRODUCCIÓ

Cellab és una marca impulsada per les empreses CIMERA (ubicada a Sant Julià de Lòria, Andorra) i CIMER (ubicada a Sant Cugat del Vallès, Espanya). Cellab realitza activitats de medicina regenerativa i biobanc de cèl·lules mare, aïllant aquestes cèl·lules mitjançant obtenció de teixit adipós i, si escau, ccriopreservant-les en un Banc de Teixits amb finalitat d'ús mèdic futur sota les mesures de qualitat requerides.

CelStem® és una solució concentrada de cèl·lules mesenquimals. Aquestes cèl·lules tenen la capacitat de renovar-se i diferenciar-se en diferents tipus cel·lulars segons la seva zona d'aplicació, aconseguint així regeneració tissular i disminució de la inflamació. Una de les fonts més riques en el nostre organisme d'aquest tipus de cèl·lules és el teixit adipós.

En l'actualitat, està creixent exponencialment el nombre d'assaigs clínics realitzats avaluant l'efecte de les cèl·lules mesenquimals pel tractament de diverses malalties. Aquests assajos, fins ara, donen suport l'ús del tractament amb cèl·lules mesenquimals procedents de teixit adipós com un tractament segur i sense efectes adversos greus. No obstant això, tot i els resultats favorables pel que fa a disminució de la inflamació i regeneració de teixit afecte, son necessaris més estudis per establir conclusions irrefutables sobre la seva eficàcia.

PROCEDIMENT

Per a l'obtenció de CelStem® és necessària l'extracció d'aproximadament 200 ml de teixit adipós abdominal, que es realitzarà mitjançant tècnica quirúrgica de lipoaspiració efectuada per un metge especialista en Medicina Estètica i Reparadora a quiròfan de Celular Clinic. Previ a la intervenció, es realitzarà extracció de sang mitjançant punció venosa per descartar qualsevol causa mèdica que contraindiqui la intervenció. En cas de contraindicació mèdica, Celular Clinic no autoritzarà l'extracció de les cèl·lules.

Les mostres de teixit adipós seran enviades a les instal·lacions de CIMERA a Sant Julià de Lòria (Andorra), on Cellab posseeix la planta productiva i els laboratoris on s'obté el producte CelStem®. Allà s'obtindrà el producte sota estrictes normes de qualitat que garanteixen el compliment de tots els estàndards necessaris per a l'obtenció d'aquest tipus de productes. L'obtenció del producte va associada a un certificat d'alliberament que garanteix el compliment de tots els requisits mínims que ha de complir. Cellab reservarà a la seva seroteca una mostra de sang per a possibles determinacions futures.

Un cop obtingut el producte CelStem® i acordat amb el metge el moment de l'aplicació, els vials amb el producte, juntament amb el certificat associat, seran enviats a Celular Clinic mantenint en tot moment les condicions òptimes de temperatura.

En cas de d'emmagatzematge exclusivament, es criopreservaran a les instal·lacions de Banc de teixit sota els estàndards de conservació.

El tractament és autòleg i únicament es pot aplicar el producte a la mateixa persona que prèviament s'ha realitzat l'extracció de teixit adipós. Per aquest motiu, l'empresa Cellab ha de disposar de la informació referent a la seva persona. Aquestes dades seran tractades de manera confidencial, segons el que estableix la Llei 15/2003, del 18 de desembre, qualificada de dades personals (BOPA 15/2003).

APLICACIÓ

Després de la preparació del producte, el metge procedirà a la seva aplicació a Celular Clínic i posterior seguiment segons criteris clínics. El procediment terapèutic consistent en infiltració de CelStem® comporta riscos menors propis de qualsevol infiltració: dolor postpunció i equimosis. Un risc infreqüent és la infecció local, que necessita tractament mèdic específic.

CONSENTIMENT

Jo, ___________________________________, amb DNI/Passaport _______________, declaro que:

- He llegit la informació continguda en aquest document.
- He pogut realitzar les preguntes pertinents i els meus dubtes han estat resoltes de manera satisfactòria.
- He estat informat detalladament del procediment que es portarà a terme, de les possibles alternatives que en fa la patologia a tractar, així com dels riscos derivats.
- He estat informat dels requisits de la normativa vigent per poder recol·lectar cèl·lules de teixit adipós i quines seran les condicions d'obtenció, transport, processament de control de qualitat, criopreservació i emmagatzematge de les mateixes.
- He estat informat que, tot i que hi ha múltiples assaigs clínics que intenten demostrar l'eficàcia del tractament amb cèl·lules mesenquimals, no hi ha conclusions irrefutables sobre això.

- Consento que Cellab realitzi les proves analítiques corresponents, i que emmagatzemi una mostra de producte final en el seu muestreoteca per a possibles anàlisis futurs.
- Consento que em recol·lectin teixit adipós per tal de ser analitzat, processat i, si escau, ser conservat sota les mesures de conservació adequades.
- Consento que se m'apliqui el producte obtingut sota estrictes normes de qualitat.
- Consento que Cellab i Celular Clinic puguin facilitar a les autoritats sanitàries competents les dades necessàries que permetin gestionar la informació relativa al procediment d'obtenció i dipòsit del meu teixit adipós d'acord amb la normativa vigent.
- Conservo la possibilitat de renunciar a aquest consentiment en qualsevol moment sense cap conseqüència.
- He estat informat pel Dr./Dra. ______________________________________

Pels motius exposats anteriorment, consenteixo que se m'apliqui el tractament CelStem® descrit en aquest document.

Escaldes-Engordany, a____ de_____________________ de _______

Signatura del pacient: Signatura del metge:

Diligència de revocació:

Jo __________________________________, amb DNI/passaport __________________, revoco el consentiment en data _________________.

Firma del pacient:

S2. Personal data transfer consent

DADES DEL PACIENT
**Nom i cognoms:**
**Telèfon:**
**Correu electrònic:**
**Passaport / DNI:**
**Direcció postal completa:**

CLÀUSULA DE CONSENTIMENT PER CESSIÓ DE DADES

De conformitat amb la **Llei 15/2003 del 18 de desembre qualificada de protecció de dades personals** i la **Llei 20/2017 del27 d'octubre de drets i deures dels usuaris i dels professionals del sistema sanitari i sobre la història clínica**, s'informa que totes les dades facilitades es tractaran amb absoluta confidencialitat i seran incorporades a un fitxer denominat HISTÒRIA CLÍNICA.

El responsable del fitxer és CIMERA SLU, amb la finalitat de prestar-li el servei mèdic sol·licitat per vostè.

Consent expressament a que les seves dades personals, incloses les de salut, puguin ser comunicades a terceres persones, en especial centres o establiments sanitaris i entitats asseguradores per a la realització de proves diagnòstiques i/o intervencions quirúrgiques necessaris, així com per al pagament de despeses i honoraris per l'atenció sanitària.

Consent expressament a que les seves dades de contacte siguin utilitzades per Celular Clinic per a recordar-li properes visites i mantenir-lo informat dels canvis que es puguin produir.

Igualment s'informa que vostè pot exercir els drets d'accés, oposició, rectificació o supressió als laboratoris Cellab, ubicats a la Borda del Germà, 14 (Sant Julià de Lòria).

Signatura i data

Escaldes-Engordany a _____ de ______________ de 20____

S3. MIBO statement

MIBO

Mesenchymal Stem Cells

Section / Topic	Item	Checklist item	Reported on Page No.
Study Design	1	Study conducted in accordance with CONSORT (RCT), STROBE (cohort, case-control or cross-sectional) or PRISMA (meta-analysis) guidelines	3
	2	Relevant institutional and ethical approval	3
Recipient details	3	Recipient demographics (including age, gender)	3
	4	Comorbidities (including underlying diabetes, inflammatory conditions, pre-existing joint pathology and smoking status)	3-4
	5	Current anti-inflammatory medications	3-4
Injury details	6	Diagnosis (including relevant grading system and chronicity)	3-4
	7	Previous treatments for current injury	4
Intervention	8	Surgical intervention described sufficiently to enable replication	4
	9	Operative findings	13-14
Donors	10	Donor age	N/A
Tissue harvest	11	Tissue harvest described sufficiently to enable replication (including anatomical source, equipment, reagents, storage media and environment)	4
	12	Time between tissue harvest and processing	4
Processing	13	Description of tissue processing that makes replication of the experiment possible (including digestion solution concentrations and volumes, duration, agitation and temperature of digestion phase, name of commercial system)	4
	14	If performed, purification described sufficiently to enable replication (including combination and concentration antibodies, equipment, method of confirming purity)	4- Sup material
	15	Yield with respect to volume of tissue processed	13-14
Cell Culture	16	If performed, cell culture described sufficiently to enable replication (including conditions, number of freeze-thaw cycles)	N/A
	17	If performed, predifferentiation described sufficiently to enable replication	N/A
MSC characteristics	18	MSC preparation and source described in title and abstract (e.g. BMMSC, ADSC)	Title-page
	19	Cellular composition / heterogeneity	N/A
	20	Immunophenotype and details of in vitro differentiation tested on batch	N/A
	21	Passage and percentage viability	
Delivery	22	MSC delivery described sufficiently to enable replication (including point of delivery, volume of suspension and media used as vehicle)	13-14
	23	If performed, details of codelivered growth factors, scaffolds or carriers	N/A
Post-operative care	24	Rehabilitation protocol sufficiently described to enable replication (including immobilisation and physical therapy)	sup material
Outcome	25	Outcome assessments include functional outcomes and recording of complications (including infection and tumour). If performed radiographic outcomes, physical examination findings, return to activities and satisfaction	5

S4. Celstem® manufacturing procedure and administration

Obtaining Celstem® requires abdominal fat liposuction. A plastic surgeon performed it under sedation and local anaesthesia in an operating room and was supervised by an anaesthetist. Intravenous cefazolin 2g was administred as infectious prophylaxis before surgery (in case of allergy, intravenous clindamycin 600mg was used). In patients with a high risk of thrombosis, subcutaneous enoxaparin 40 mg was prescribed for the procedure day. The electrocardiogram, blood pressure, and pulse oximetry were monitored during the intervention.

Cleaning with povidone-iodine was undertaken to minimise the risk of contamination of the adipose tissue with skin saprophytes. Moreover, sterile transparent film dressings were placed on the incision areas. The fat tissue extraction was done using 3 to 4 mm cannulas with 10 cc syringes and 2 to 3 cc pressure directly placed in the transfusion bag to avoid fat manipulation. The extraction amount varied according to each patient's availability, with minimum extraction of 150 to 200 mL. After closing the small incisions with sutures, dressings were placed over them, and an abdominal girdle that compressed the area between the submammary region and mid-thighs was prescribed. The extracted material, which contained oil, adipose tissue, blood, local anaesthetic, and stromal vascular fraction (SVF), was immediately transported (within one hour) to Cellab Laboratory under standardised refrigeration conditions. A traceability certificate was filled out to ensure its correct conservation.

The product's preparation was carried out exclusively in a laboratory cleanroom, following the American Association of Blood Banks (AABB) standards hygiene and biological safety conditions. The preparation started with an exhaustive washing of the fat extracted with saline solution in order to remove both residues of blood or lidocaine and adrenaline used in the extraction process. Then, the fat was concentrated and mixed with the enzymatic digestion solution (consisting of collagenase [NORDMARK, Ref: N0002779] at 0.75 mg/mL). The mixture was incubated at 37 °C and stirred for 30 to 40 minutes. When this process was finished, collagenase was inactivated through human albumin and then again thoroughly washed with saline solution to remove the remnants of undigested fat and collagenase. A concentrated cell suspension in a reduced volume

was obtained. The final product was analysed to determine cellularity, cell yield, viability, and colony-forming power, and a manufacturing certificate was issued.

Once prepared, the product returned under appropriate refrigeration conditions to Celular Clinic, where the infiltration procedure was carried out. A certificate of correct traceability correctly was again completed to ensure its proper conservation.

The treatment injection was carried out on the same day within six hours after the extraction. Under sterility measures, ultrasonography-guided intraarticular injection of Celstem® was performed in the affected knee or knees. Previous synovial fluid aspiration was performed if required. Once the procedure was completed, rehabilitation recommendations and clinical follow-up information were provided.

S5. Post-procedure recommendations

Post-Procedure Recommendations

1. **Compression Garment**:
   - Wear the abdominal compression garment for approximately 20 days post-procedure.
   - Do not remove it during the first three days under any circumstances.
   - From the third day onward, remove it only for hygiene purposes. In some cases, it can be removed at night after 15 days.

2. **Wound Care**:
   - Maintain the incision sites clean and dry at all times.

3. **Medication**:
   - Avoid non-steroidal anti-inflammatory drugs during the first two weeks, as they may interfere with treatment efficacy. Pain management should be limited to paracetamol or tramadol if necessary.

4. **Activity Restrictions**:
   - Relative rest is advised for the first three days, including using one or two crutches if needed.
   - After one week, and once sutures are removed and wounds healed, active mobility exercises are encouraged. Water-based activities may also be initiated to reduce joint impact.
   - After 15 days, physical activity can gradually increase, provided it remains low-impact.
   - Full resumption of sports activities is generally recommended after two months, subject to individual evaluation by the treating physician onn the basis of the specific sport.
